# Exercise snacks and cognitive health in older adults: mechanisms, implementation, and public health relevance

**DOI:** 10.3389/fpubh.2026.1850516

**Published:** 2026-06-16

**Authors:** Xianjun Liu, Yibing Xia, Xinman Gao, Rui Wang

**Affiliations:** 1 School of General Education, Dalian University of Technology, Dalian, China; 2 Dalian School of Economics and Trade, Dalian, China

**Keywords:** cognitive aging, exercise snacks, implementation, intermittent physical activity, older adults

## Abstract

As a major contributor to disability and loss of independence in older adults, cognitive decline poses growing public health challenge in aging societies worldwide. Although pharmacological therapies, cognitive training, and conventional exercise may provide benefits in selected contexts, their real-world impact is often limited by modest efficacy, poor long-term adherence, or difficulties with integration into daily life. Exercise snacks, defined as brief and structured bouts of activity performed intermittently across the day, have emerged as a potentially feasible and low-burden approach for supporting cognitive health in later life. In this Perspective, we examine the potential relevance of exercise snacks to cognitive health in older adults. We argue that their value may lie not only in possible physiological effects, but also in their flexibility and implementability in everyday routines. However, direct evidence linking exercise snacks to cognitive outcomes in older adults remains limited, and much of the available literature is heterogeneous in design, short in duration, and focused on acute or indirect outcomes. We therefore distinguish direct cognitive evidence from broader mechanistic inferences and discuss several plausible pathways, including neurobiological, metabolic, and psychosocial processes. We also highlight major research gaps and outline priorities for future work, including clearer definitions, longer-term trials, more sensitive cognitive outcomes, and real-world implementation strategies. If these issues are addressed, exercise snacks may become a practical strategy for supporting cognitive resilience in later life.

## 1 Introduction

Age-related cognitive decline is a major contributor to disability, loss of independence, and reduced quality of life in older adults. Across the spectrum from normal cognitive aging to mild cognitive impairment (MCI) and dementia, preserving cognitive health has become an urgent public health priority in rapidly aging societies (1). Dementia represents the most severe end of this continuum and illustrates the scale of the challenge, with an estimated 55 million people affected worldwide in 2019 and a projected increase to 139 million by 2050 (2). However, although dementia highlights the magnitude of the problem, the present Perspective focuses more broadly on cognitive health in community-dwelling older adults, including those experiencing normal cognitive aging or at increased risk of decline. This broader public health context underscores the need for feasible, low-burden strategies that can be integrated into daily life, including brief and intermittent forms of physical activity such as exercise snacks.

Regular physical activity has been consistently shown to support cognitive function across the lifespan, particularly in older adults. Meta-analyses indicate a dose–response relationship, with higher frequency, duration, and intensity of exercise generally associated with greater improvements in global cognition, memory, and executive function (3, 4). These benefits are observed both in cognitively healthy individuals and those with mild cognitive impairment, highlighting the broad relevance of exercise for brain health. Exercise snacks, defined as brief, intermittent bouts of activity throughout the day, may leverage these mechanisms by providing accessible, low-burden opportunities to stimulate neurocognitive processes, potentially supporting attention, memory, and executive function in older adults. Current approaches to maintaining or improving cognitive function in older adults include pharmacological therapy (5, 6), cognitive training (7), exercise intervention, and psychotherapy (8). However, these methods usually have problems such as limited efficacy and poor adherence. Although these strategies may offer benefits in specific contexts, their real-world impact is often limited by modest efficacy, heterogeneity of response, poor long-term adherence, or difficulties with implementation in daily life. Among these approaches, exercise is widely recognized as a promising non-pharmacological strategy for supporting cognitive health. However, many older adults find it difficult to sustain conventional structured exercise programs because of health limitations, time constraints, low motivation, lifestyle barriers, or the perceived burden of formal training (9, 10). These challenges suggest that, beyond asking whether exercise is beneficial, it is equally important to consider how exercise can be made more feasible and sustainable in everyday life for older adults.

Within this context, exercise snacks have emerged as a potentially useful behavioral format. Although definitions vary across studies, the term generally refers to brief bouts of physical activity performed intermittently across the day, often in home- or community-based settings (11). Recent reviews have also noted that terminology in this field remains inconsistent, partly overlapping with related concepts such as accumulated exercise, sedentary breaks, and other short-bout activity patterns. Therefore, the value of exercise snacks should not be assumed simply on the basis of novelty. Rather, their potential significance lies in the possibility that short, distributed bouts of activity may reduce participation barriers, fit more naturally into daily routines, and broaden access to movement opportunities for older adults who may not engage in conventional exercise programs (12).

At present, however, direct evidence linking exercise snacks to cognitive outcomes in older adults remains limited. This limitation reflects several sources of heterogeneity, including inconsistent definitions of exercise snacks, variation in bout duration, intensity, frequency, timing, and modality, differences in target populations, and the use of diverse cognitive outcome measures (13). In addition, some available studies focus primarily on physiological, metabolic, or functional outcomes rather than cognition itself, making it difficult to determine whether observed benefits are exercise-snack-specific cognitive effects or broader effects of increased physical activity. Recent population-based findings have provided additional support for a possible association between exercise snacks and better cognitive performance in older adults, but causal inference remains premature (14).

**Figure 1:**
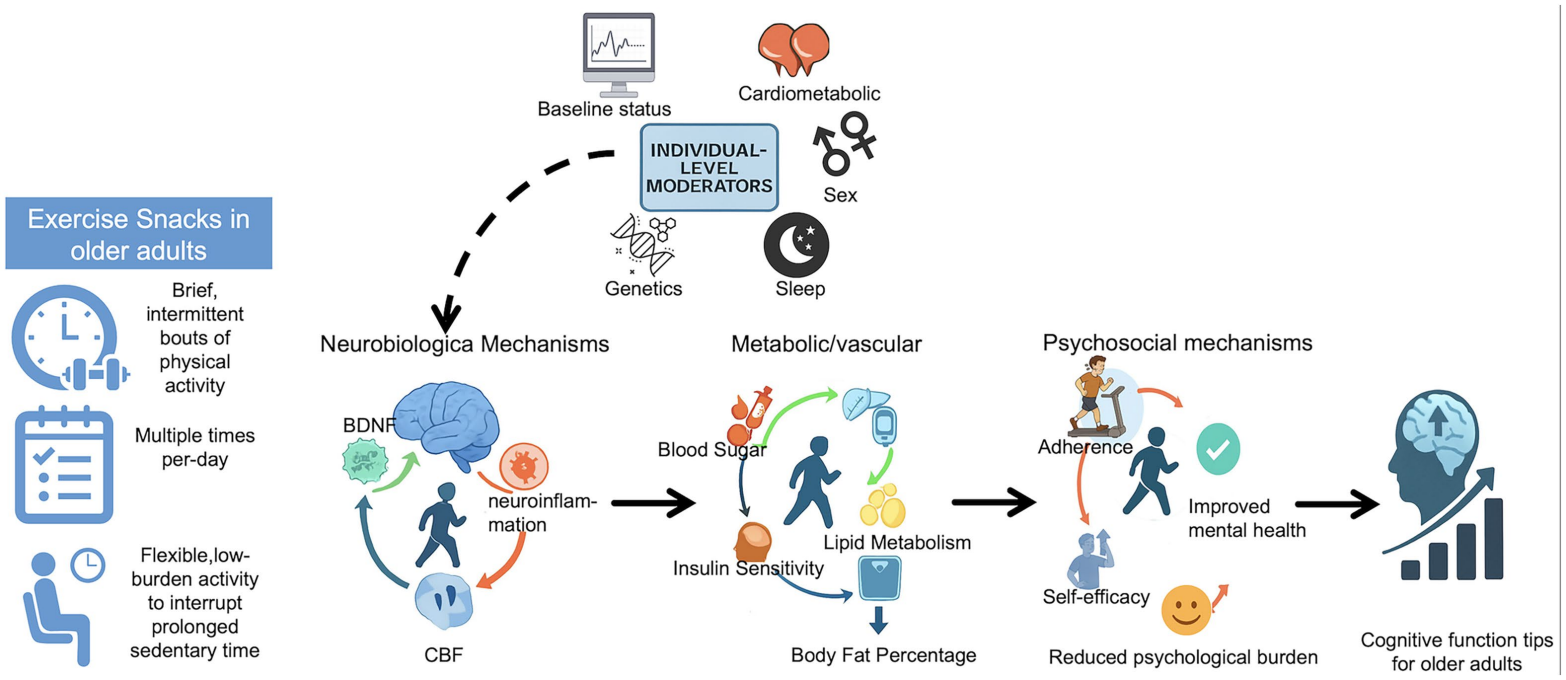
Exercise snacks and cognitive health in older adults: mechanistic pathways.

In this Perspective, we synthesize the current evidence on exercise snacks and cognitive health in older adults, distinguish direct cognitive evidence from broader mechanistic inferences derived from exercise research, and propose a provisional conceptual framework through which exercise snacks may influence cognitive outcomes via neurobiological, metabolic, vascular, and psychosocial pathways. We also highlight key limitations in the current literature and identify priorities for future research, including clearer conceptual definitions, better characterization of target populations, more rigorous cognitive endpoints, and pragmatic trials that examine feasibility, sustainability, and real-world scalability. Figure 1 presents this conceptual framework, with particular attention to individual-level moderators and implementation-related factors.

## 2 Characteristics of exercise snacks

Exercise snacks are best understood as brief, purposeful, and structured bouts of exercise performed intermittently across the day. Typical modes include stair climbing, brisk stepping, sit-to-stand repetitions, and body-weight resistance exercises. Existing studies commonly use very short bouts lasting approximately 1–5 min, although some brief-bout or accumulated activity protocols extend toward 10 min (77, 78). Therefore, we regard bouts of approximately 1–5 min as typical of exercise snacks, while using ≤10 min as a pragmatic upper boundary to distinguish this format from conventional continuous exercise sessions. These thresholds should not be interpreted as strict biological cutoffs. Rather, they are operational boundaries intended to improve conceptual clarity, facilitate comparison across studies, and preserve the defining feature of exercise snacks: short and repeatable bouts that can be embedded into daily routines.

Intensity should be interpreted as a prescription variable rather than a strict defining feature of exercise snacks. The defining characteristics of exercise snacks are their brief, purposeful, structured, intermittent, and repeatable format, rather than a fixed intensity threshold (11). For relatively healthy older adults, moderate-to-vigorous relative intensity may be appropriate when the goal is to elicit cardiorespiratory, cerebrovascular, or metabolic stimulation (15). In supervised or research settings, intensity may be described using physiological indicators such as percentage of heart-rate reserve, percentage of maximal heart rate, or METs. In home- and community-based settings, more practical tools such as rating of perceived exertion, the talk test, and functional tolerance may be more feasible (16). For frail, sedentary, or multimorbid older adults, light-to-moderate bouts may still qualify as exercise snacks when they are purposeful, structured, repeated, and adapted to functional capacity. However, simple incidental movement or unplanned posture changes should not automatically be classified as exercise snacks.

Intensity may also shape the dominant mechanisms through which exercise snacks influence cognitive health. Higher-intensity snacks may be more likely to induce acute changes in arousal, catecholamine activity, cerebral perfusion, neurotrophic signaling, and cardiometabolic demand (17). By contrast, light-to-moderate snacks may operate more through sedentary interruption, postprandial metabolic regulation, affective response, self-efficacy, adherence, and functional engagement (18). Therefore, exercise-snack prescriptions should be individualized according to baseline fitness, comorbidity, cognitive status, safety, and implementation context, and future studies should report intensity clearly and examine whether cognitive responses differ according to intensity level and target population.

Importantly, exercise snacks should be distinguished from several related but non-identical concepts. They are not equivalent to simple interruptions of sedentary time, incidental lifestyle movement accumulated across the day, vigorous intermittent lifestyle physical activity embedded in daily living, or conventional continuous exercise sessions of longer duration (19). In practice, exercise snacks may be delivered using scheduled or situational cues, such as fixed intervals during the day, after prolonged sitting, or around meal times. However, these triggers are better understood as implementation features rather than defining characteristics of the concept. To reduce conceptual ambiguity and improve consistency in future research, Box 1 summarizes the operational definition and scope adopted in this Perspective.

BOX 1 **“Exercise snacks” for seniors—operational definition and scope**

- Operational Definition: Exercise snacks are brief, purposeful, and structured bouts of exercise performed intermittently across the day, typically lasting 1–5 min per bout. A ≤10 min threshold is used here as a pragmatic upper boundary to distinguish this format from conventional continuous exercise, rather than as a strict biological cutoff.

- Intensity: Intensity is a prescription variable rather than a strict defining criterion. It may be monitored using heart-rate reserve, percentage of maximal heart rate, METs, perceived exertion, the talk test, or functional tolerance, and should be adapted to older adults’ health status and functional capacity.

- Distinctions: Exercise snacks are distinct from simple sedentary breaks, incidental lifestyle movement, vigorous intermittent lifestyle physical activity in daily living, and conventional continuous exercise sessions.

- Delivery and triggers: They may be delivered using scheduled or situational cues, such as fixed intervals, after prolonged sitting, or around meal times.

The following sections synthesize the current evidence on exercise snacks and cognitive health in older adults and discuss the potential mechanisms through which this approach may influence cognitive outcomes.

## 3 Evidence and mechanisms of exercise snacks in cognitive decline prevention

Current evidence directly linking exercise snacks to cognitive outcomes in older adults remains limited. Existing studies are mostly acute or short-term, involve relatively small samples, and vary substantially in exercise dose, timing, modality, and outcome assessment. Accordingly, the present Perspective does not argue that exercise snacks have already been proven superior to traditional continuous exercise. Rather, we suggest that exercise snacks deserve attention as a feasible and implementation-oriented format of physical activity that may be relevant to cognitive health in later life. Recent population-based evidence has further strengthened this rationale. In a cross-sectional analysis of older adults from NHANES 2011–2014, exercise snacks were associated with better cognitive performance, and an inverted U-shaped relationship was observed, suggesting that this movement pattern may be relevant to cognitive health but still requires prospective and interventional confirmation.

### 3.1 Comparative efficacy vs. traditional continuous exercise

Currently, head-to-head comparisons of older adults with cognitive outcomes as the primary endpoint and under the premise of weekly equivalence (or controlled energy/total MVPA) are almost nonexistent. Limited studies have shown that, under weekly equivalence, short-duration, frequent, intraday “exercise snacks” do not differ significantly from continuous long-duration exercise in multiple health outcomes (20–22). This conclusion applies only to equivalence and not to comparisons of unmatched doses.

Consistent with this, public health and population evidence shows that even with a lower total volume than the standard continuous exercise regimen, low-dose, fragmented physical activity still provides substantial benefits (such as lower all-cause mortality and depression risk) and is significantly better than inactivity (23–26). However, the above studies are not equivalence comparisons and do not constitute evidence of “equivalence” with long-duration exercise. Meanwhile, authoritative guidelines have removed the ≥10 min/segment threshold, clarifying that any duration of moderate-intensity physical activity of moderate or higher intensity can be included in the weekly recommended intake, providing methodological and policy basis for the prescription of “short-duration × high-frequency, intraday-dispersed” (20). Furthermore, multiple existing research reports show that snacking solutions have higher compliance and completion rates, lower churn and preparation burdens, and, under the premise of weekly equivalents, these implementation differences may translate into greater real-world net benefits.

### 3.2 Direct cognitive outcomes in older adults

Current direct evidence linking exercise snacks to cognitive outcomes in older adults remains limited. Most available studies are acute or short-term, involve relatively small samples, and show substantial heterogeneity in exercise dose, timing, modality, and cognitive assessment. Recent systematic reviews suggest that short-duration, frequent physical activity may produce small but detectable acute benefits in cognitive performance, particularly in attention, inhibitory control, working memory, and cognitive flexibility, with reported effect sizes in the small range (27). Another review also reported generally positive directional effects on executive function, attention, working memory, and cognitive flexibility; however, quantitative synthesis was limited by marked between-study heterogeneity, and evidence for longer-term cognitive benefit remains insufficient (28). In older adult samples, evidence from randomized crossover and short-term intervention studies suggests that exercise snacks may lead to modest improvements, or at least non-inferior trends, in executive function, attention, and processing speed over the course of the same day or across several weeks (29, 30). However, not all studies have reported significant changes, indicating that the observed effects may depend on exercise dose, intervention timing, cognitive task selection, and statistical power (31). Importantly, some studies cited in this area do not include cognitive outcomes as primary endpoints, instead focusing on physiological, metabolic, or functional indicators that may be relevant to brain health but do not directly demonstrate cognitive benefit. In the present Perspective, such studies should therefore be regarded as supportive but indirect evidence, rather than as direct proof of cognitive effectiveness.

Another important limitation is the choice of cognitive outcome measures. Some studies rely primarily on global screening tools such as the MMSE or MoCA, which may be insufficiently sensitive to detect subtle short-term changes in specific cognitive domains. In particular, exercise snacks may be more likely to influence executive function-related domains, including inhibitory control, working memory, and cognitive flexibility, as well as attention and processing speed. Future studies would therefore benefit from incorporating standardized cognitive tasks or composite outcome measures that are more sensitive to these domain-specific changes, rather than relying solely on global cognitive screening tools (32, 33).

### 3.3 Mechanistic pathways underlying exercise snacks and cognitive protection in older adults

The potential mechanisms linking exercise snacks to cognitive health should be interpreted with caution. Rather than simply viewing sports snacks as a “shortened version” of traditional exercise prescriptions, it is better to understand them as a lifestyle intervention that restructures the daily time structure of older adults. Short, repetitive, and fragmented activities are embedded in a day that is originally filled with sedentary behavior. Each 2–5 min activity will produce a small “disturbance” in terms of neural activity, metabolic load, and emotional experience. When such disturbances occur multiple times in a day and accumulate over a long period on a weekly or monthly scale, they may be transformed into macroscopic adaptations: the brain receives blood flow and nutrients more regularly, the body processes glucose and lipids more efficiently, and the individual’s sense of control over their own body and emotional state become more stable.

From this integrative perspective, neurobiological, metabolic, and psychosocial mechanisms are not three parallel pathways that are isolated from each other, but different facets of the same process: neural and metabolic changes provide the biological basis for cognitive maintenance (34), while psychosocial self-efficacy, mood improvement, and compliance determine whether such behavior can be maintained in the real world in the long term. This section will explore these three complementary pathways, synthesizing evidence directly from cognitive outcomes and related research in the fields of cardiovascular, metabolic and mental health (35), and propose several action hypotheses that can be translated and verified, rather than giving definitive causal conclusions.

To provide a clearer overview of this mechanistic framework and its public health implications, Table 1 summarizes the current state of evidence, identified limitations, and future research and implementation strategies across neurobiological, metabolic, psychosocial, and integrated pathways.

**Table 1:** Mechanistic insights into exercise snacks and cognitive health in older adults: evidence, limitations, and future research/implementation strategies.

Mechanistic pathway	Current state of evidence	Identified problems/limitations	Future research and implementation strategies
Neurotrophic signaling / BDNF	Short-term exercise may acutely increase BDNF levels, and BDNF is relevant to neuronal survival, synaptic plasticity, and memory-related processes. In the context of exercise snacks, repeated brief bouts may provide intermittent neurotrophic stimulation.	Most evidence is extrapolated from broader exercise studies rather than exercise-snack-specific trials. Acute BDNF responses do not necessarily translate into immediate or sustained cognitive improvement, and inter-individual variability remains substantial.	Future studies should measure BDNF and domain-specific cognitive outcomes within the same design, while distinguishing acute biomarker responses from long-term cognitive adaptation. From a public health perspective, studies should prioritize feasible and minimally burdensome protocols that can be delivered in home or community settings rather than only in laboratory environments.
Cerebral blood flow / cerebrovascular regulation	Brief aerobic or intermittent activity may acutely increase cerebral perfusion and may be associated with short-term improvements in attention, working memory, processing speed, and executive performance.	Evidence is inconsistent regarding the magnitude, duration, and cognitive relevance of CBF changes. Direct evidence that repeated exercise snacks produce sustained cerebrovascular adaptation remains limited.	Future research should combine cognitive testing with feasible neurovascular measures, such as near-infrared spectroscopy, transcranial Doppler, or cerebral perfusion imaging where available. Public health-oriented studies should also examine whether simpler field-based indicators, such as blood pressure, mobility, and vascular risk profiles, can help identify older adults most likely to benefit.
Neuroinflammatory regulation	Chronic low-grade neuroinflammation is associated with cognitive decline, and regular exercise may reduce inflammatory markers such as TNF-α and IL-6. Exercise snacks may plausibly influence this pathway if repeated over weeks or months.	Direct evidence that exercise snacks modulate neuroinflammatory pathways in older adults is scarce. Acute immune responses to brief bouts may occur, but their cognitive relevance remains unclear.	Future trials should include inflammatory biomarkers alongside cognitive and functional outcomes. For public health translation, research should focus on higher-risk groups, such as older adults with cardiometabolic vulnerability, frailty, or elevated dementia risk, to determine whether exercise snacks can serve as a low-burden preventive strategy.
Metabolic regulation	Exercise snacks may acutely attenuate postprandial glucose excursions, reduce sedentary-related metabolic exposure, and improve glycemic regulation. Longer-term repeated bouts may support insulin sensitivity and cardiometabolic health.	Evidence is stronger for metabolic outcomes than for direct cognitive outcomes, but findings remain heterogeneous for lipid metabolism, adiposity, and body composition. The link between metabolic improvement and cognitive benefit remains largely indirect.	Future studies should test whether improvements in glucose regulation, insulin sensitivity, lipid metabolism, or adiposity mediate cognitive changes. Public health strategies should examine how exercise snacks can be integrated into daily routines, especially after meals or during prolonged sitting, and whether they can be incorporated into community health programs, primary care advice, and chronic disease prevention initiatives.
Psychosocial and behavioral mechanisms	Exercise snacks may reduce perceived burden, improve immediate confidence and affective response, and support adherence by fitting into daily routines. Repeated participation may strengthen self-efficacy and sustained physical activity engagement.	Current evidence mainly supports feasibility and acceptability rather than direct cognitive benefit. Adherence is influenced by supervision, motivation, intervention complexity, cultural context, and participant characteristics.	Future research should assess self-efficacy, mood, perceived burden, habit formation, and adherence as potential mediators. Public health implementation should test culturally adaptable, low-cost, home-based, and community-based models, with particular attention to older adults who are inactive, socially isolated, frail, or have limited access to structured exercise facilities.
Integrated pathway / cumulative adaptation	Exercise snacks may operate through repeated acute responses that accumulate into longer-term neurobiological, metabolic, and psychosocial adaptations. Their public health relevance may depend on feasibility and sustainability rather than large immediate cognitive effects.	Existing studies are mostly acute or short-term, with small samples and heterogeneous protocols. Direct long-term evidence linking multi-level mechanisms to sustained cognitive and functional benefit remains limited.	Future research should prioritize long-term pragmatic RCTs, standardized but adaptable protocols, sensitive domain-specific cognitive outcomes, real-world functional measures, and implementation outcomes such as reach, adoption, adherence, maintenance, equity, and cost-effectiveness. These designs would help determine whether exercise snacks can become a scalable strategy for cognitive health promotion in aging populations.

#### 3.3.1 Neurobiological mechanisms

At the neurobiological level, brain-derived neurotrophic factor (BDNF), cerebral blood flow (CBF), and neuroinflammatory regulation are among the most plausible pathways linking physical activity to cognitive aging. These indicators are closely related to brain plasticity, energy supply, and the neural immune environment, and may therefore be relevant to the maintenance of cognitive function in older adults. However, direct evidence that exercise snacks specifically influence these pathways in ways that translate into cognitive benefit remains limited. Accordingly, the following discussion should be understood as identifying plausible and testable mechanisms, rather than established causal explanations. Importantly, these neurobiological pathways may operate across different time scales. Acute effects may occur during or shortly after a single exercise-snack bout, whereas long-term adaptations may require repeated exposure over weeks or months.

BDNF is a key neurotrophic factor involved in neuronal survival, synaptic plasticity, and memory-related processes (35, 36). At an acute time scale, short-term exercise interventions may rapidly increase BDNF levels, including in older adults (30, 37). In the context of exercise snacks, a single brief bout may therefore induce a transient neurotrophic response that could contribute to short-term changes in neural excitability, arousal, and cognitive performance. However, the relationship between acute BDNF responses and cognitive improvement remains uncertain. An acute increase in BDNF does not necessarily translate into immediate or sustained cognitive benefit, and individual responses may vary substantially across age, health status, baseline fitness, and exercise intensity (38). From a long-term perspective, the potential relevance of BDNF may lie less in the effect of a single brief exercise bout and more in the cumulative influence of repeated neurotrophic stimulation. For exercise snacks, the key hypothesis is not that one 2–5 min bout substantially changes brain function, but that recurring brief bouts performed across the day and sustained over weeks or months may repeatedly stimulate the neurotrophic environment. Over time, such repeated stimulation may help support synaptic plasticity and compensatory processes in the aging brain. Nevertheless, direct evidence that exercise snacks produce sustained BDNF-mediated cognitive benefits in older adults remains limited, and this pathway should therefore be interpreted as biologically plausible but not yet established.

CBF is fundamental for maintaining brain metabolism and function. At an acute time scale, short-duration aerobic or intermittent activity may increase whole-brain or region-specific cortical perfusion within a time window of several minutes to tens of minutes, and such changes may be associated with short-term improvements in executive function, attention control, working memory, and processing speed (39–41). In the context of exercise snacks, these acute cerebrovascular responses may be particularly relevant because brief bouts can interrupt prolonged sitting and provide repeated short periods of increased cerebral perfusion across the day. However, existing findings are not entirely consistent in terms of effect size, duration, or cognitive relevance, and some studies have not observed a clear CBF–cognition linkage (42). These findings suggest that acute increases in CBF may be a plausible but insufficient explanation for cognitive benefits on their own. From a longer-term perspective, the key issue may not be how long a single increase in CBF lasts, but whether repeated episodes of brief activity can improve the overall cerebrovascular environment over time. For exercise snacks, a pattern of “multiple short-duration perfusion” may help counteract prolonged periods of sedentary-related low perfusion and low metabolic demand across the day. If sustained over weeks or months, this repeated stimulation may contribute to more stable cerebrovascular regulation and a more favorable metabolic background for older adults, particularly those at elevated risk of cognitive decline. Nevertheless, direct evidence that exercise snacks induce long-term cerebrovascular adaptations leading to sustained cognitive benefits remains limited, and this mechanism should be considered a testable hypothesis rather than a confirmed causal pathway.

Neuroinflammatory regulation may also be relevant, particularly from a longer-term perspective. Chronic low-grade neuroinflammation is widely regarded as an important contributor to cognitive decline and neurodegenerative disease, partly through the activation of microglia and astrocytes and the sustained elevation of inflammatory mediators. Although a single bout of physical activity may induce transient immune and cytokine responses, the cognitive relevance of these acute responses remains uncertain. More plausibly, repeated exercise snacks performed over weeks or months may contribute to a gradual reduction in chronic inflammatory burden, thereby creating a more favorable neural environment for cognitive maintenance. Exercise interventions have been associated with reductions in inflammatory markers such as tumor necrosis factor-*α* (TNF-α) and interleukin-6 (IL-6), alongside broader benefits for cognitive and brain health (43). However, direct evidence that exercise snacks specifically modulate neuroinflammatory pathways in older adults remains scarce. At present, this mechanism should therefore be considered biologically plausible but still largely inferential in the context of exercise snacks.

#### 3.3.2 Metabolic mechanisms

Metabolic regulation represents another plausible pathway linking exercise snacks to cognitive health in older adults. Insulin resistance, impaired glycemic control, dyslipidemia, and excess adiposity are all associated with a higher risk of cognitive decline, suggesting that metabolic health is closely intertwined with cognitive aging (44–47). From this perspective, the potential relevance of exercise snacks lies not only in increasing total energy expenditure, but also in their ability to redistribute movement across the day and thereby modify the temporal pattern of metabolic exposure, particularly during prolonged sedentary periods and after meals See Figure 1 and Table 2. These metabolic processes may operate across both acute and longer-term time scales: brief bouts may acutely modify postprandial and sedentary-related metabolic exposure, whereas repeated exercise snacks over weeks or months may contribute to more sustained improvements in metabolic regulation.

**Table 2:** Key studies of exercise snacks or sedentary-break interventions and cognitively relevant outcomes in older adults.

Study	Design & sample	Snack/break protocol vs. comparator	Outcomes (cognitive/other)	Main finding
Wheeler et al. (30)	3-arm acute crossover RCT; community-dwelling older adults	(A) Prolonged sitting with brief light–moderate walking breaks; (B) single continuous bout of moderate exercise; vs. uninterrupted sitting (single laboratory visit)	Working memory and executive function: small acute ↑ vs. sitting; hemodynamic measures (systemic/cerebral blood flow)	Breaking up sitting or a single exercise bout produced small acute improvements in executive function vs. prolonged sitting.
Cunha et al. (29)	Acute crossover RCT; older adults	Breaking up prolonged sitting with light–moderate single-task or dual-task walking bouts vs. uninterrupted sitting (single-day exposure)	Global cognition and executive tasks: better performance with walking, especially dual-task; CBF ↑ with breaks vs. sitting	Walking breaks, especially dual-task walking, improved cognition and CBF compared with uninterrupted sitting.
Maasakkers et al. (31)	Acute crossover trial; older adults at risk for cognitive decline	Sedentary behavior with brief physical and/or mental activity breaks vs. continuous sedentary condition (short-term, hours)	Attention, executive function and other domains; cerebral hemodynamics	Continuous sitting worsened CBF and some cognition, while brief physical/mental breaks mitigated these effects.
Li et et al. (40)	Pilot RCT; sedentary middle-aged workers	Workplace-integrated exercise snacks (brief, frequent bouts during workday) vs. usual sedentary work routine (multi-week intervention)	Attention, working memory; feasibility and acceptability indicators	Workplace exercise snacks were feasible and showed trends toward better cognitive performance in sedentary workers.
Zhou et al. (55)	RCT; sedentary obese adults	Exercise snacks used to break up prolonged sitting across the day vs. prolonged sitting / standard lifestyle control (≈12-week intervention)	Cognition: not assessed; other: body composition, plasma metabolomics profiles	Exercise snacks improved body composition and metabolomic cardiometabolic markers, despite no cognitive outcomes being assessed.
Liang et al. (59)	Mixed-method feasibility study; self-isolating older adults during COVID-19	Home-based strength and Tai Chi “exercise snacking” delivered remotely; short bouts accumulated across the day vs. no structured exercise/usual care (~?-week program)	Cognition: not assessed; other: feasibility, acceptability, adherence, physical function	Remote strength and Tai Chi snacking in older adults was highly feasible, acceptable and well adhered to.
Liang et al. (61)	Exploratory RCT; community-dwelling older adults	12-week progressive home-based strength and Tai Chi exercise snacking (multiple short bouts/day on most days) vs. control or minimal intervention	Cognition: not reported; other: muscle strength, physical function, adherence, qualitative experience	Progressive strength and Tai Chi snacking improved physical function with good adherence in community-dwelling older adults.

At an acute time scale, exercise snacks may be especially relevant when performed after meals or during prolonged sedentary periods. Brief bouts of activity can increase skeletal muscle activity and glucose utilization, attenuate postprandial glucose excursions, and reduce fluctuations in circulating free fatty acids, thereby limiting repeated exposure to an adverse metabolic environment across the day. This may be particularly important for older adults, who often experience longer sedentary periods and greater vulnerability to impaired glucose regulation. Several studies suggest that brief and repeated bouts of activity can improve insulin sensitivity and glycemic regulation, especially among individuals with low physical activity levels or poorer metabolic health (48, 49). However, these acute metabolic responses should not be interpreted as direct evidence of cognitive improvement. Rather, they may represent short-term physiological changes that reduce daily metabolic stress and provide a plausible indirect pathway through which exercise snacks could support brain health.

From a longer-term perspective, repeated exercise snacks may contribute to more sustained metabolic adaptations. Traditional exercise interventions such as aerobic exercise and resistance exercise have been widely acknowledged to improve insulin sensitivity (50), glycemic control (51), lipid metabolism, and body fat percentage (52). Compared with the neurobiological pathways discussed above, the metabolic effects of exercise snacks are supported by somewhat more direct evidence, particularly in relation to glycemic regulation and cardiometabolic health. If brief bouts are repeated consistently across days and weeks, they may help improve insulin sensitivity, reduce repeated postprandial metabolic stress, and support a more favorable cardiometabolic profile. These longer-term changes may be especially relevant because vascular and metabolic dysfunction are important contributors to cognitive aging and may indirectly affect brain maintenance through cerebrovascular, inflammatory, and energetic pathways.

Nevertheless, evidence for longer-term metabolic adaptations remains heterogeneous. Although some studies have reported favorable effects on lipid metabolism, others have not observed significant changes in triglyceride or high-density lipoprotein cholesterol levels (49). Similarly, findings regarding body fat percentage and weight-related outcomes are mixed. Some studies suggest that exercise snacks may reduce adiposity, including abdominal fat, whereas others report little or no measurable change, possibly because of differences in baseline health status, exercise intensity, cumulative dose, or intervention duration, or adherence (53). Taken together, these findings suggest that the metabolic effects of exercise snacks are promising but context-dependent.

In relation to cognitive health, the most plausible interpretation is that both acute and longer-term metabolic effects contribute indirectly to brain maintenance rather than acting as isolated cognition-specific mechanisms. Acute improvements in postprandial or sedentary-related metabolic exposure may reduce short-term physiological stress, whereas longer-term improvements in insulin sensitivity, glycemic control, lipid regulation, and adiposity may reduce chronic vascular and metabolic burden. Thus, the metabolic pathway provides an important but still incomplete explanation for the potential cognitive relevance of exercise snacks in later life. Future studies should therefore examine metabolic biomarkers and cognitive outcomes together, in order to clarify whether improvements in metabolic regulation mediate any sustained cognitive benefits of exercise snacks in older adults.

#### 3.3.3 Psychosocial mechanisms

Psychosocial and behavioral pathways may be particularly important in understanding the potential relevance of exercise snacks to cognitive health in older adults. For many older individuals, the key challenge is not only whether exercise can improve health under ideal conditions, but whether an exercise routine can be initiated and sustained in everyday life. In this context, factors such as self-efficacy, affective response, perceived burden, and adherence may be especially influential (54, 55). These psychosocial processes may operate across both acute and longer-term time scales: brief bouts may produce immediate affective and motivational responses, whereas repeated participation may shape self-efficacy, adherence, and sustained engagement over time.

Compared with conventional continuous exercise, which often requires dedicated time, preparation, and in some cases access to specific facilities or equipment, exercise snacks may reduce both practical and psychological barriers to participation by breaking activity into brief, flexible segments. At an acute time scale, completing a brief and manageable bout of activity may provide an immediate sense of success, reduce exercise-related intimidation, and improve perceived control. Such short-term psychological responses may be particularly relevant for older adults who have low confidence, limited exercise experience, or concerns about fatigue and safety. This format may lower the threshold for initiation, reduce the perceived difficulty of exercise, and facilitate integration into daily routines, thereby making long-term engagement more achievable in real-world settings (56).

From a longer-term perspective, the psychosocial value of exercise snacks may lie less in any single session and more in their potential to support repeated participation over time. Repeated successful completion of short activity bouts may strengthen self-efficacy, enhance perceived control, and increase willingness to remain physically active. Over time, these experiences may help transform brief movement opportunities into more stable behavioral routines. In this way, exercise snacks may contribute to cognitive health indirectly by supporting sustained physical activity engagement, rather than by producing immediate cognitive improvement through psychosocial mechanisms alone.

Available adherence data provide some support for this interpretation. For example, supervised exercise-snack interventions have shown adherence rates comparable to those reported for moderate-intensity continuous training and high-intensity interval training, whereas in unsupervised settings exercise snacks may achieve relatively higher adherence (57). However, these findings should be interpreted cautiously, as adherence can be influenced by study design, supervision, participant motivation, and intervention complexity. Thus, rather than demonstrating the superiority of exercise snacks over other exercise formats, the current evidence more reasonably suggests that exercise snacks may represent a feasible and scalable option for promoting physical activity among older adults and other insufficiently active populations (58, 59).

In addition to adherence, exercise snacks may also influence cognitive health indirectly through emotional well-being. At an acute time scale, short and manageable activity bouts may improve mood, perceived energy, and confidence after completion, which could support willingness to repeat the behavior. Some studies also suggest that brief activity formats may help reduce fatigue, anxiety, depressive symptoms, or discomfort, thereby supporting broader mental well-being (60). From a longer-term perspective, repeated positive affective experiences may reinforce motivation, improve exercise-related self-efficacy, and reduce the perceived burden of remaining active (61). Nevertheless, direct evidence linking these psychosocial changes specifically to long-term cognitive outcomes remains limited. At present, the psychosocial pathway is best understood as a plausible and potentially important indirect mechanism through which exercise snacks may enhance real-world engagement and, in turn, contribute to cognitive maintenance in later life.

#### 3.3.4 Integrated view: linking acute responses with long-term adaptation

Taken together, the available evidence suggests that the potential relevance of exercise snacks to cognitive health in older adults is unlikely to depend on any single pathway or a single time scale. Rather, neurobiological, metabolic, and psychosocial processes may interact across acute and longer-term levels. At an acute time scale, individual exercise-snack bouts may induce transient changes in neurotrophic signaling, cerebral perfusion, postprandial metabolic exposure, mood, perceived energy, and executive performance (28, 29). However, these immediate responses should not be interpreted as direct evidence of sustained cognitive protection.

From a longer-term perspective, the potential value of exercise snacks may lie in the repeated activation of these acute responses over time. Repeated short bouts of activity may contribute to cognitive maintenance by supporting a more favorable neurotrophic and cerebrovascular environment, improving day-to-day metabolic regulation, and enhancing adherence, self-efficacy, and emotional well-being (62). In this sense, exercise snacks may be best understood as a cumulative behavioral strategy: small physiological and psychosocial responses produced by brief bouts may become more meaningful when they are repeated consistently across days, weeks, or months.

At present, however, this integrated framework should still be regarded as provisional. Most existing studies are short-term, involve relatively small samples, and report effects that are generally small to moderate in magnitude. Moreover, the available evidence is stronger for some pathways than for others, and direct long-term evidence linking these multi-level changes to sustained cognitive benefit remains limited (29, 31). Therefore, the acute effects of exercise snacks and their potential long-term adaptations should be interpreted as related but distinct phenomena. Acute improvements in attention, executive performance, mood, or metabolic regulation may provide useful mechanistic signals, but they do not by themselves establish durable cognitive benefit.

Nevertheless, when considered in the context of everyday life, the potential value of exercise snacks may lie less in producing large immediate changes on conventional cognitive scales and more in helping older adults maintain cognitive and functional stability over time. Such perspective is particularly important because the meaningful outcomes of cognitive health interventions in later life extend beyond test scores alone. They also include everyday capacities such as medication management, financial decision-making, household organization, social participation, and the preservation of independence and quality of life.

Even when measurable cognitive effects appear modest, a low-burden and implementable activity format that helps older adults remain engaged, active, and functionally capable may still have substantial public health significance. Viewed in this way, exercise snacks may represent a feasible, daily, and potentially scalable strategy for supporting cognitive resilience in later life. This integrated perspective also provides a useful conceptual basis for future longitudinal and pragmatic studies that simultaneously assess biomarkers, domain-specific cognitive outcomes, and real-world functional measures (63).

### 3.4 Modality-specific considerations for exercise snacks

Exercise snacks should not be regarded as a homogeneous intervention simply because they share a brief and intermittent format. Different exercise modalities may trigger distinct physiological and psychosocial pathways and may therefore have different implications for cognitive outcomes in older adults. Aerobic exercise snacks, such as brief walking, stair climbing, or stepping bouts, may primarily act through acute increases in cerebral blood flow, cardiorespiratory activation, vascular regulation, and arousal. Evidence from aerobic exercise studies in older adults suggests that aerobic training may improve selected cognitive domains and cerebrovascular regulation, and longer-term aerobic interventions may increase cerebral blood flow and improve vascular function (64, 65). These responses may be particularly relevant to attention, processing speed, working memory, and other executive function-related domains.

Resistance-based exercise snacks, such as sit-to-stand repetitions, squats, calf raises, or elastic-band exercises, may operate through somewhat different pathways. Their cognitive relevance may be linked less to immediate cerebrovascular responses and more to skeletal muscle activation, glucose uptake, insulin sensitivity, muscle strength, and functional capacity. Resistance training has been associated with improved overall cognitive function in older adults with different cognitive status, while resistance exercise-snacking interventions have shown feasibility, safety, and acceptability in community-dwelling older adults (13, 66). For older adults, especially those with frailty risk or low physical function, these effects may indirectly support cognitive health by improving mobility, reducing cardiometabolic burden, and preserving independence in daily life.

Body–mind exercise snacks, including brief Tai Chi, Baduanjin, yoga-based movements, or breathing-coordinated balance exercises, may engage additional attentional and psychosocial pathways. These modalities combine low-to-moderate physical demand with postural control, body awareness, breathing regulation, and attentional focus. Systematic reviews suggest that Tai Chi may enhance cognitive function in older adults, particularly executive function, and may exert benefits through its combined physical, cognitive, and meditative components (67). Recent work on Tai-chi snacking also suggests that brief home-based formats may be acceptable and feasible for older adults, although physical function and cultural context may shape uptake and experience (12, 59). These modalities may therefore be particularly relevant to cognitive flexibility, attentional control, dual-task performance, mood regulation, and stress reduction. However, direct comparative evidence across aerobic, resistance, and body–mind exercise snacks remains limited. Future studies should therefore examine not only the dose, frequency, and timing of exercise snacks, but also the modality-specific mechanisms and cognitive domains most likely to respond to each type of activity.

## 4 Research gaps and insufficient existing evidence

Despite growing interest in exercise snacks as a potentially feasible strategy for supporting cognitive health in older adults, the current evidence base remains preliminary and incomplete. First, most available studies have focused on acute effects or short-term interventions, often with relatively small sample sizes and substantial heterogeneity in intervention design, cognitive assessment, and outcome reporting (28). This limits comparability across studies and makes it difficult to draw robust conclusions regarding the magnitude, consistency, and durability of cognitive benefit.

Second, key features of exercise-snack interventions remain insufficiently standardized. Definitions vary across studies, and important parameters such as bout duration, intensity, frequency, timing, and cumulative dose are not reported consistently (68). As a result, the dose–response relationship remains poorly understood, and it is still unclear which exercise-snack patterns are most relevant for different cognitive outcomes or target populations. Greater conceptual and methodological consistency will be essential for advancing the field.

Third, evidence in populations at elevated risk of cognitive decline remains especially limited. Although some short-term studies suggest that exercise snacks may have favorable cognitive or physiological effects, there is still a lack of systematic long-term research in older adults with mild cognitive impairment, cardiometabolic vulnerability, frailty, or increased risk of neurodegenerative disease (31). This restricts the generalizability and clinical relevance of current findings and highlights the need for future studies in more diverse and better-characterized older populations.

Fourth, current research provides limited guidance on how exercise snacks should be tailored to individual differences in health status, functional capacity, cognitive profile, or motivational readiness. Future work should move beyond one-size-fits-all approaches and examine how exercise-snack prescriptions can be adapted for robust, pre-frail, and frail older adults, as well as for those with differing levels of cognitive risk or exercise tolerance. Such personalization will be particularly important for real-world implementation and long-term adherence.

Finally, the broader intervention context has not yet been adequately explored. Most existing studies focus on exercise snacks as a single-component intervention, whereas their potential value may be enhanced when combined with cognitive training, psychological support, social engagement, or behavior-change strategies (63). In addition, digital health tools, wearable monitoring, and remote prompting systems may offer new opportunities to improve adherence, personalization, and scalability, but these possibilities remain underexamined (49).

Taken together, these gaps suggest that future research should prioritize longer-term and pragmatic study designs, clearer intervention definitions, more sensitive and functionally meaningful cognitive outcomes, and greater attention to individual heterogeneity and implementation in daily life. Addressing these issues will be essential to determine whether exercise snacks can evolve from a promising concept into a practical and scalable approach for supporting cognitive resilience in later life.

## 5 Outlook

### 5.1 Clinical implications and future directions

Exercise snacks may represent a promising and low-burden approach for supporting cognitive health in older adults, particularly from the perspective of feasibility, adherence, and integration into daily life (13). However, the current evidence base remains insufficient to support definitive clinical recommendations. Most existing studies are short-term, small in scale, and heterogeneous in intervention design, making it difficult to determine the durability, magnitude, and generalizability of cognitive effects (28). Accordingly, the next stage of research should move beyond proof-of-concept studies toward more rigorous and clinically relevant evaluation.

A key priority is the development of more standardized and better-characterized intervention protocols. Future studies should report and test the effects of bout duration, intensity, frequency, timing, and cumulative dose more systematically in order to clarify dose–response relationships and identify which exercise-snack patterns are most appropriate for different outcomes and populations (13). For older adults, this will require particular attention to individual variation in functional capacity, comorbidity, and cognitive risk. In this regard, exercise-snack prescriptions may need to be tailored according to health status and tolerance, especially for individuals with mild cognitive impairment, frailty, cardiometabolic vulnerability, or elevated risk of neurodegenerative disease.

From a clinical translation perspective, exercise snacks could be considered as brief movement prescriptions in primary care, geriatric clinics, memory clinics, rehabilitation services, community health programs, and long-term care settings (20). Rather than being presented as a stand-alone treatment for cognitive decline, they may be more appropriately introduced as a low-burden adjunct to existing healthy aging, chronic disease management, and dementia-risk reduction strategies (69). In clinical practice, such prescriptions would require basic safety screening, attention to fall risk and comorbidities, individualized progression, and clear guidance on when, how often, and at what intensity brief bouts should be performed.

Another major priority is the generation of long-term causal evidence. Existing work has largely focused on acute responses, cross-sectional associations, or short intervention windows, whereas the maintenance of cognitive health is a gradual process that unfolds over months or years (28). High-quality randomized controlled trials are therefore needed to determine whether exercise snacks can causally improve cognitive performance, daily functioning, and independence over time. Such trials should include adequately powered samples, clearly defined intervention protocols, appropriate control or comparator groups, sufficient follow-up duration, preregistered outcomes, and blinded cognitive assessment where feasible (70). They should also incorporate sensitive and functionally meaningful cognitive measures, particularly for executive function, inhibitory control, working memory, cognitive flexibility, attention, and processing speed, rather than relying solely on global screening tools. In addition, pragmatic RCTs conducted in home, community, primary care, or senior-service settings would be especially valuable for determining whether any cognitive benefits can be sustained and implemented under real-world conditions (63).

Mechanistic research also remains essential. Although neurotrophic signaling, cerebral perfusion, metabolic regulation, and psychosocial adaptation all represent plausible pathways, relatively few studies have directly examined whether exercise snacks influence these mechanisms in ways that translate into long-term cognitive benefit. Future studies should combine cognitive testing with biomarkers, neuroimaging, vascular measures, metabolic indicators, and behavioral assessments in order to clarify which pathways are most relevant, for whom, and under what conditions.

Overall, future work should follow a staged research agenda. The immediate priority is to establish causal evidence through adequately powered RCTs with sensitive domain-specific cognitive outcomes. The next priority is to develop standardized but adaptable protocols that specify duration, intensity, frequency, timing, modality, and progression. Subsequent studies should clarify mechanisms by combining cognitive testing with biomarkers, neurovascular measures, metabolic indicators, and psychosocial assessments. Finally, pragmatic trials should evaluate whether effective protocols can be implemented, maintained, and scaled in real-world clinical and community settings (71).

The clinical and public health value of exercise snacks may depend not only on efficacy, but also on implementation. Future research should therefore address how exercise snacks can be embedded into real-life care and community settings, how adherence can be maintained over time, and how psychological or practical barriers to participation can be reduced. Multicomponent intervention models that combine exercise snacks with cognitive training, psychological support, social engagement, or digital prompting tools may be especially worthwhile to test (69). If these issues are adequately addressed, exercise snacks may evolve from an emerging concept into a practical and scalable strategy for supporting cognitive resilience in later life.

### 5.2 Applications and implementation strategies

The potential value of exercise snacks lies not only in their physiological relevance, but also in their implementability in everyday settings. Their short duration and flexible format make them particularly suitable for integration into community- and home-based routines, where older adults often face practical barriers to participating in conventional structured exercise. Rather than requiring dedicated sessions, specialized facilities, or substantial time commitment, exercise snacks may be incorporated into daily life through brief, manageable bouts of movement, thereby offering a lower-burden option for increasing physical activity in later life (13, 58).

This practical relevance may be especially important in settings such as community health programs, home-based support models, nursing homes, and senior wellness services. In such contexts, exercise snacks could be embedded into existing care routines or daily schedules to promote regular movement without imposing excessive physical or psychological burden. However, their value in these settings should not be assumed automatically. Effective implementation will depend on whether interventions are acceptable, feasible, and sustainable for older adults with differing levels of functional capacity, cognitive risk, and support needs. Future implementation studies should therefore evaluate not only efficacy, but also reach, adoption, feasibility, acceptability, fidelity, maintenance, scalability, and cost-effectiveness (63). These outcomes are essential for determining whether exercise snacks can move beyond controlled research settings and become a sustainable public health strategy for older adults.

Digital health tools may further expand the implementation potential of exercise snacks. Wearable devices, smartphone applications, remote prompting systems, and virtual coaching models may help older adults monitor activity, receive feedback, and maintain structured participation outside clinical or supervised environments. These approaches may be particularly useful for overcoming barriers related to mobility, distance, or limited access to in-person programs (72). At the same time, digital implementation should be approached cautiously. Digital tools may unintentionally widen inequalities if older adults have limited digital literacy, restricted internet access, financial barriers to device ownership, privacy concerns, or low confidence in using technology (73). Technology fatigue and data-security concerns may also reduce long-term engagement. Therefore, digital exercise-snack interventions should be accompanied by non-digital alternatives, such as printed prompts, telephone follow-up, caregiver-supported routines, or community-based guidance.

Adherence remains one of the central challenges in real-world implementation. Even when exercise snacks are brief and flexible, older adults may still face psychological barriers, uncertainty about safety, low confidence, or limited motivation to remain active over time. For this reason, implementation strategies should go beyond simple prescription and include behavior-support components such as achievable goal setting, prompting systems, feedback, encouragement, and social support. Cultural and contextual factors may also shape how exercise snacks are perceived and adopted, suggesting that implementation models should be adapted to local expectations, routines, and preferences rather than applied uniformly across populations.

Sustainability should not be assumed simply because exercise snacks are brief. Long-term maintenance will depend on whether brief bouts can be linked to stable daily cues, embedded into existing routines, adapted as functional capacity changes, and supported by caregivers, peers, or community workers when needed. Low-cost delivery, minimal equipment requirements, and flexible progression may also be important for maintaining participation over time.

Finally, the successful translation of exercise snacks into practice will likely require interdisciplinary collaboration. Input from exercise science, gerontology, psychology, rehabilitation, digital health, and community care may help develop interventions that are not only physiologically appropriate, but also behaviorally realistic and context-sensitive. In the future, exercise snacks may be most effective when integrated into broader multicomponent programs that combine movement with cognitive training, psychological support, health education, or social engagement (69). From this perspective, the key challenge is not simply whether exercise snacks can be prescribed, but how they can be implemented in ways that are acceptable, sustainable, and meaningful in the daily lives of older adults.

### 5.3 Policy and public health considerations

The broader translation of exercise snacks into practice will depend not only on further clinical research, but also on support from public health systems and policy frameworks. If exercise snacks are to become a meaningful component of strategies for supporting cognitive health in older adults, they will need to be incorporated into health promotion, healthy aging, and chronic disease management initiatives at the community and population levels. In this context, the relevance of exercise snacks lies in their potential to offer a low-burden and potentially scalable movement option that can be embedded into existing services for older adults.

Policy support may be particularly important for creating the conditions under which exercise snacks can be implemented consistently across different care and community settings. Public health agencies, community organizations, primary care services, and older-adult support programs may all play a role in promoting brief, feasible activity formats as part of broader healthy aging strategies. However, such integration should be evidence-informed and context-sensitive, rather than based on the assumption that exercise snacks will automatically be adopted or effective across all populations.

Equity and accessibility should therefore be considered from the outset. Older adults differ substantially in socioeconomic resources, housing conditions, neighborhood safety, digital literacy, physical function, cognitive status, social support, and access to health or community services. Exercise-snack interventions should be designed to remain feasible for individuals who are frail, socially isolated, digitally excluded, or living in resource-limited settings. This may require low-cost, equipment-free options, non-digital delivery materials, caregiver or community-worker support, and culturally appropriate activity choices.

Economic considerations may also influence whether exercise snacks are taken seriously at the system level. Future research should therefore evaluate not only cognitive and functional outcomes, but also feasibility, cost-effectiveness, and potential downstream effects on healthcare utilization and long-term care burden (74, 75). Cost-effectiveness analyses should also consider equity impacts, including whether low-burden exercise-snack programs can reach older adults who are underserved by conventional facility-based exercise programs. Such evidence may help determine whether exercise snacks can provide value as part of population-level approaches to promoting healthy aging and reducing the burden associated with cognitive decline.

Public awareness and health communication will also be important for broader uptake. Educational efforts may help older adults and caregivers better understand that meaningful movement does not always require long, formal, or high-intensity exercise sessions. In this sense, exercise snacks may be particularly compatible with public health messaging that emphasizes achievable, acceptable, and sustainable forms of activity. Their impact may be further strengthened when aligned with broader initiatives in mental health, chronic disease management, and social participation (76).

Ultimately, the public health importance of exercise snacks will depend on whether they can be translated into interventions that are not only biologically plausible, but also practical, acceptable, and sustainable in real-world settings. This will require coordinated efforts across research, policy, community services, and healthcare systems. If these conditions are met, exercise snacks may contribute to a broader shift toward more implementable and inclusive strategies for supporting cognitive resilience in later life.

## 6 Conclusion

Exercise snacks may represent a promising and low-burden approach for supporting cognitive health in older adults. Rather than being viewed simply as a shortened version of conventional exercise, they may be better understood as a feasible and implementation-oriented movement format that can be embedded into daily life. From this perspective, their potential relevance lies not only in possible physiological and cognitive effects, but also in their capacity to reduce participation barriers and support sustained engagement in physical activity among older adults.

At present, however, the evidence base remains preliminary. Existing studies are mostly acute or short-term, involve relatively small samples, and vary substantially in intervention design, target populations, and outcome measures. Moreover, although several neurobiological, metabolic, and psychosocial pathways may plausibly explain how exercise snacks could contribute to cognitive maintenance, direct evidence linking these mechanisms to sustained cognitive benefit in older adults remains limited. Exercise snacks should therefore be regarded not as an established clinical solution, but an emerging and testable strategy that warrants further rigorous investigation.

Nevertheless, the concept remains important from both clinical and public health perspectives. In aging societies, cognitive decline increasingly threatens independence, quality of life, and care systems, and there is a clear need for intervention formats that are not only effective in principle, but also realistic and sustainable in everyday settings. In this context, exercise snacks may offer a useful complement to traditional exercise approaches by emphasizing feasibility, flexibility, and long-term adherence.

Future research should now move beyond short-term proof-of-concept studies and focus on standardized intervention protocols, long-term and pragmatic trials, mechanism-informed designs, and real-world implementation across diverse older populations. If these issues are adequately addressed, exercise snacks may evolve from a promising concept into a practical and scalable strategy for supporting cognitive resilience in later life.

## Data Availability

The original contributions presented in the study are included in the article/supplementary material, further inquiries can be directed to the corresponding author.
